# Sensory Nerve‐Derived CGRP Controls Osteoclastogenesis by Limiting Macrophage Bioenergetics in Bone Repair

**DOI:** 10.1002/advs.202518303

**Published:** 2026-03-01

**Authors:** Jiaying Liu, Ting Zhang, Yuqing Mu, Lili Li, Ju Jin, Kevin J Dudley, Wendong Gao, Donglin Cai, Fuhua Yan, Lan Xiao, Yin Xiao

**Affiliations:** ^1^ School of Medicine and Dentistry Griffith University Gold Coast Queensland Australia; ^2^ Institute for Biomedicine and Glycomics Griffith University Gold Coast Queensland Australia; ^3^ Australia Centre for Precision Health and Technology Griffith University Gold Coast Queensland Australia; ^4^ Nanjing Stomatological Hospital Affiliated Hospital of Medical School Institute of Stomatology Nanjing University Nanjing Jiangsu China; ^5^ Clem Jones Centre for Neurobiology and Stem Cell Research Griffith University Brisbane Queensland Australia; ^6^ Central Analytical Research Facility School of Biology and Environmental Science Queensland University of Technology Brisbane Queensland Australia

**Keywords:** bone repair, CGRP, immunometabolism, neuro–immune interaction, osteoclastogenesis, osteoimmunology, regenerative medicine

## Abstract

Bone healing is a tightly orchestrated, multiphase process that requires coordinated interactions between immune cells and skeletal cells. Sensory nerves act as intrinsic effectors of the inflammatory response, whose role in osteoimmunology during healing remains poorly defined. Using a bone healing model with sensory denervation, it's shown that sensory nerves protect bone repair by suppressing excessive osteoclastogenesis. During the acute inflammatory phase, sensory nerves are upstream regulators of macrophage activation. At the molecular level, calcitonin gene‐related peptide (CGRP), a sensory neuron–derived neuropeptide, is identified to modulate macrophage activation by restricting key functions such as migration, phagocytosis, and pro‐inflammatory cytokine production. Importantly, CGRP rapidly constrains adenosine triphosphate (ATP) synthesis and mitochondrial respiration in activating macrophages, accompanied by downregulation of genes associated with oxidative phosphorylation and mitochondrial complex components. Following the metabolic alterations, macrophages exposed to CGRP show attenuated osteoclastogenic capacity, with decreased secretion of multiple key factors that support osteoclast differentiation and survival. Together, these findings indicate a neuro–immune–metabolic axis in bone healing, where sensory nerve–derived CGRP influences macrophage bioenergetics and thereby contributes to osteoimmunoligical regulation. It emphasizes the potential of incorporating sensory signals into therapeutic strategies, particularly those targeting immunometabolism in bone regeneration.

## Graphical abstract

Sensory nerves help bones heal. This study shows that the neuropeptide CGRP, released from sensory nerves, slows down macrophage energy production, preventing excessive bone breakdown. By revealing this nerve–immune–metabolism connection, the work provides new insight into how the body balances bone repair and opens doors to targeted treatments.

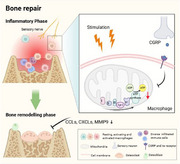

## 1 Introduction

Bone is a rigid but dynamically remodeling organ that supports the skeletal system and provides the foundation for physical activities [1]. Even though bone has endogenous regenerative capacity, delayed, inadequate, or failed bone healing still occurs and significantly affects global populations [2, 3]. In 2019, the Years Lived with Disability (YLD) caused by fractures alone were reported at 25.8 million among all ages, despite many other causes of bone defects [4]. In fact, successful bone healing is often challenged by multiple factors, as the process is highly orchestrated and relies on the integration of diverse biological signals [1, 2]. Despite advances in regenerative medicine, approaches that enable efficient and precise control of bone healing remain limited, largely due to insufficient understanding of the intrinsic biological signals that govern the bone healing cascade [5].

Bone healing is initiated and driven by inflammation, which establishes the reparative microenvironment in the early phase [6]. At the onset of the healing cascade, necrotic debris, clot formation, and potential microbial invasion collectively trigger an inflammatory response [6]. Guided by chemotactic signals, innate immune cells are rapidly recruited to the wound site, where they clear cell debris and infectious organisms while simultaneously regulating bone forming/remodeling cells (e.g. osteoblasts and osteoclasts) through amplified downstream signals [5]. The reciprocal interaction between immune cells and bone cells laid the foundation for the field of “osteoimmunology” [7]. In the context of bone repair, for instance, excessive release of pro‐inflammatory cytokines such as TNFα and IL‐1β suppresses osteoblast differentiation while promoting osteoclastic survival and bone resorption [7, 8]. Thus, the well‐coordinated inflammatory response is decisive for bone repair outcomes. However, the inflammatory response is shaped not only by many pathological conditions (e.g. diabetes) but also by physiological intrinsic signals (e.g. neural molecules, hormones, lipid mediators) that remain underexplored [9, 10, 11, 12, 13

Insights from neuroimmunology suggest that sensory neurons are more than passive detectors of stimuli, but also actively regulate innate immunity [14, 15, 16]. Once activated, sensory neurons generate electrical impulses (action potential) that travel towards nearby peripheral nerve terminals via the axon reflex [14]. This rapid mechanism enables sensory neurons to release neuropeptides such as calcitonin gene‐related peptide (CGRP) directly into the affected region, thereby modulating neighboring cells. However, studies have reported diverse immunomodulatory effects of sensory neurons, ranging from pro‐inflammatory to anti‐inflammatory, depending on the specific neural molecules involved and the biological context (e.g., different organs and disease models) [16, 17, 18, 19, 20, 21, 22, 23]. This variability highlights that the immunomodulation of sensory nerves is likely tissue‐specific and shaped by distinct physiological and pathological conditions.

Despite these advances, the role of sensory nerves in osteoimmunology, the immune–bone cell interactions that drive the sequential dynamics of bone healing, remains unclear. While mineralized bone tissue itself lacks sensory innervation, nerve fibers are present in the periosteum, Haversian canals, and bone marrow [19, 24, 25]. As efficient intrinsic regulators of immune responses, sensory nerves could play a significant role in modulating immune cell behavior and thereby influence the bone healing cascade as the upstream signaling elements. Therefore, the present study aims to uncover the role of sensory nerves in osteoimmunology during bone healing. These insights not only advance fundamental understanding of bone biology but may also inspire innovative clinical strategies to enhance regenerative outcomes.

## 2 Results

### 2.1 Sensory Nerve Ablation Impairs Bone Healing with Exacerbated Osteoclastogenesis

**FIGURE 1:**
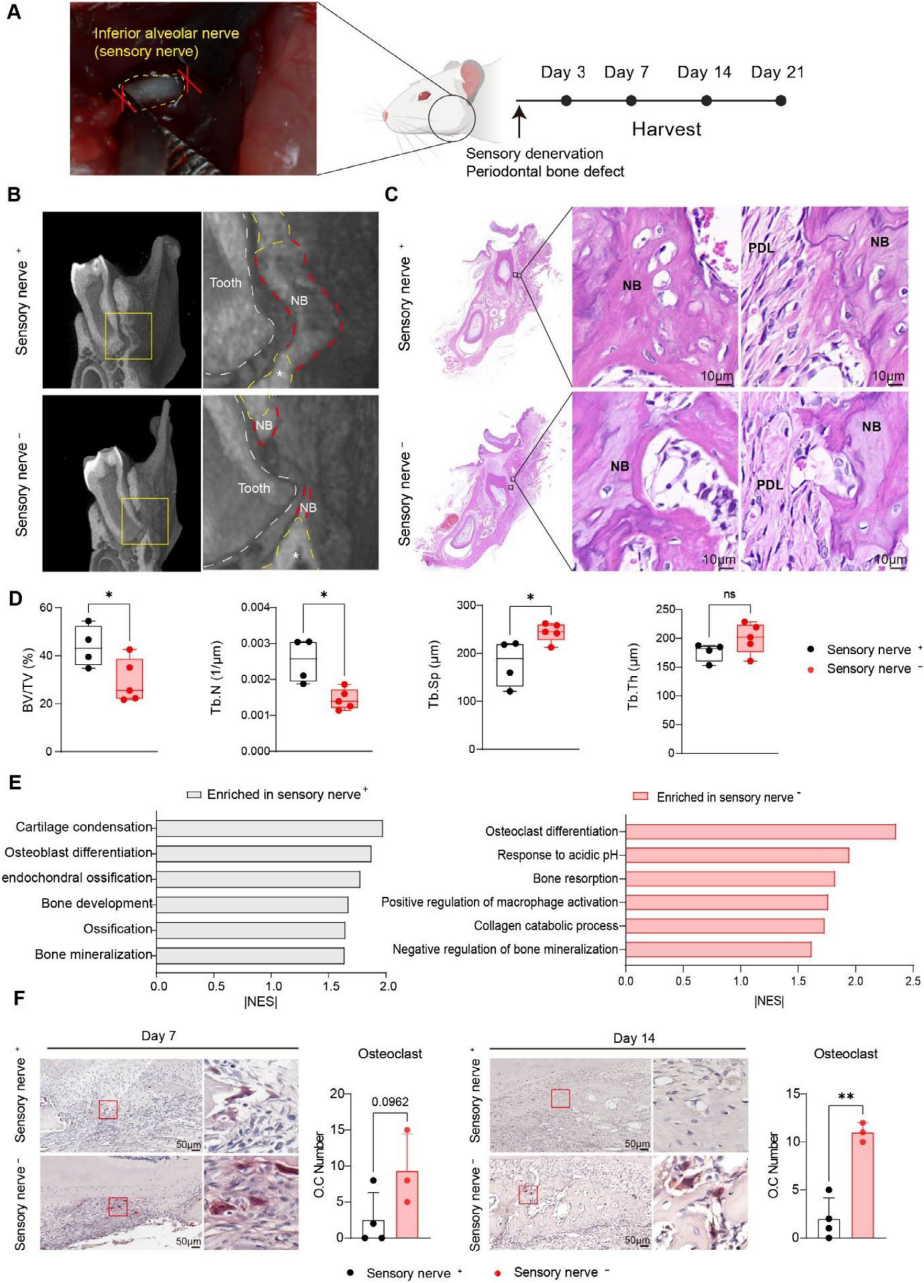
Sensory denervation impairs bone healing by inducing excessive osteoclast activity. (A) Schematic illustration of the experimental design regarding animal work, with the picture (left) showing the exposed sensory nerve prior to transection. (B) Representative images from micro‐CT 3D reconstruction showing new bone (NB, red) and pre‐existing bone (*, yellow) after 3 weeks of healing in the sensory nerve^+^ group and the sensory nerve^−^ group. (C) Representative H&E staining after 3 weeks of healing in the sensory nerve+ group and the sensory nerve^−^ group, showing the overview of rat mandibles, followed by high‐magnification images exhibiting newly formed bone (NB) and Sharpey's fibers at the periodontal ligament (PDL)‐newly formed bone (NB) interface. (D) Quantitative micro‐CT analysis of bone volume fraction (BV/TV), trabecular number (Tb. N) and higher trabecular separation (Tb. Sp) in sensory nerve^+^ and sensory nerve^−^ groups after 3 weeks of healing. ^*^ *P < *0.05 by Student's *t* test, *n* = 4 to 5 per group. (E) Gene set enrichment analysis (GSEA) of RNA‐seq data regarding healing tissue from day 7 post‐surgery, exhibiting biological processes enriched in each group. *P* < 0.05, *n* = 4 per group. NES, normalized enrichment score. (F) Representative images of TRAP staining identifying osteoclasts in bone defects on day 7 and day 14, corresponding quantification of TRAP^+^ osteoclast numbers at each time point in each group. ^**^ *P < *0.01 by Student's *t* test, *n* = 3 to 4 per group. Dots represent individual animals. Data are presented as means ± SD.

To investigate the general role of sensory nerves in bone repair, we established a bone defect model combined with sensory nerve ablation by performing transection of the inferior alveolar nerve, which provides sensory innervation to the mandible (Figure 1A). Simultaneously, a standardized periodontal bone defect was created on the mandible. After 3 weeks of healing, sensory nerve ablation markedly impaired bone regeneration, resulting in reduced bone volume and compromised bone quality (Figure 1B). Micro‐computed tomography (CT) measurements revealed significantly lower bone volume fraction (BV/TV), decreased trabecular number (Tb. N) and increased trabecular separation (Tb. Sp) in sensory‐denervated bone defects compared with controls (Sensory nerve^+^) (Figure 1D). Histology confirmed these findings, showing inferior bone quality and comparatively disorganized collagen fibers at the interface between the periodontal ligament (PDL) and newly formed bone (NB) in the sensory‐denervated group (Figure 1C).

To gain mechanistic insights, RNA‐sequencing (RNA‐seq) of healing tissues at day 7 revealed distinct transcriptional features between sensory nerve^+^ and sensory nerve^−^ groups. By comparing gene set enrichments from healing tissue with or without sensory innervation, tissues with intact sensory innervation were enriched in biological processes associated with osteoblast differentiation, ossification and bone mineralization (Figure 1E). In contrast, sensory‐denervated tissues exhibited enrichment in pathways linked to osteoclast differentiation, bone resorption, and collagen catabolism (Figure 1E). Given the central roles of osteoblasts and osteoclasts in bone remodeling, we further quantified their numbers by histological analysis. Although a reduction in RUNX2^+^ cell numbers was observed in sensory nerve^−^ group at day 14 (Figure S1), the increase in osteoclast numbers was more prominent and appeared as early as day 7, persisting through day 14 (Figure 1F). Collectively, these findings suggest that impaired bone healing following sensory nerve ablation is predominantly attributable to exacerbated osteoclastogenesis.

### 2.2 Sensory Nerves Regulate Macrophage Behavior in the Acute Inflammatory Phase

**FIGURE 2:**
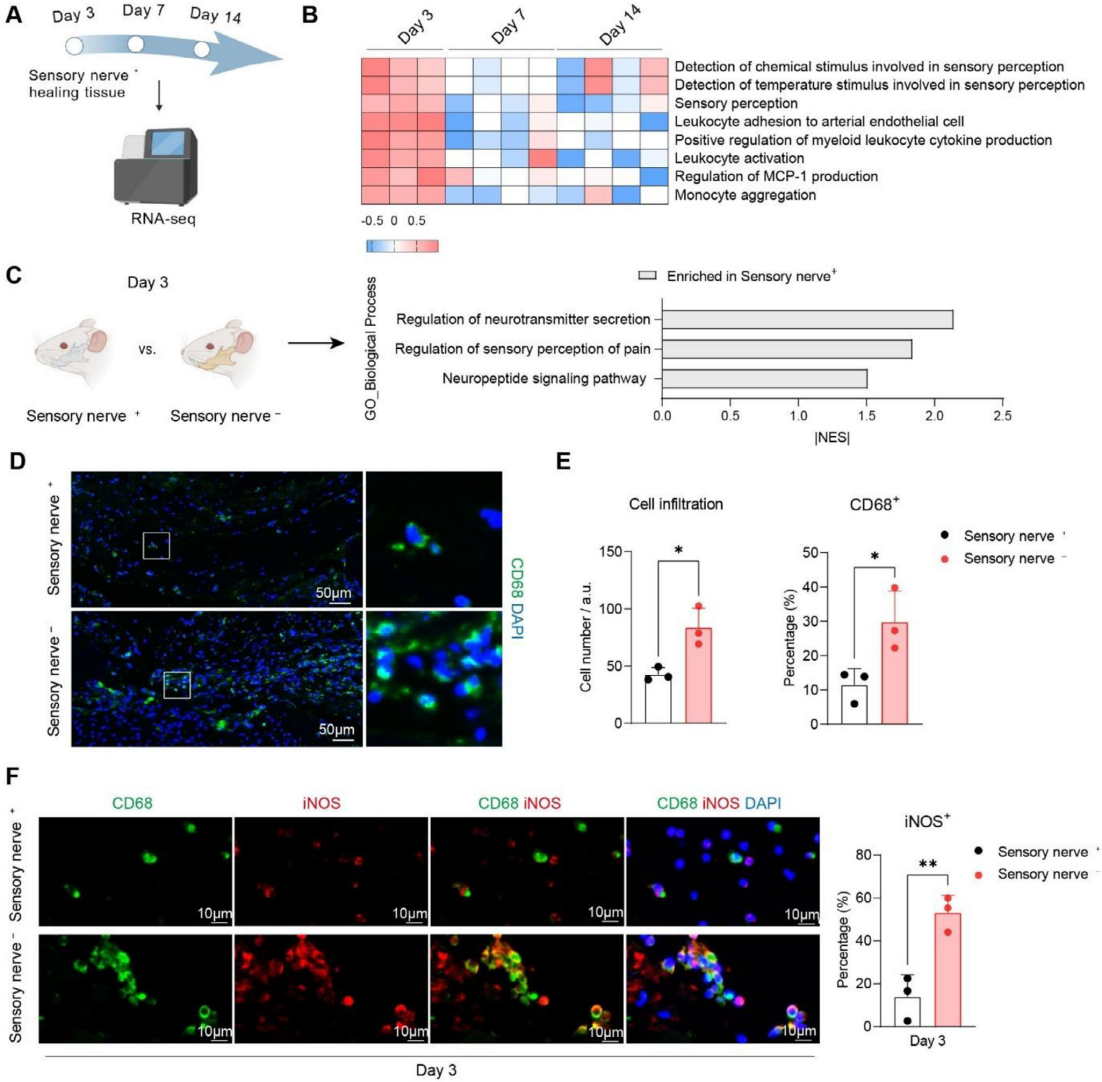
Sensory nerve‐derived signals regulate macrophage behavior in the acute inflammatory phase of bone repair. (A) Schematic illustration showing healing tissue in normally innervated bone at different time points was collected for RNA‐seq. (B) Heatmap of gene set variation analysis (GSVA) exhibiting gene sets distinctively enriched at day 3, compared to day 7 and day 14 during normally innervated bone healing. *P* < 0.05, *n* = 3 to 4 per group. The color represents normalized enrichment score. (C) Schematic illustration showing comparison of healing tissue on day 3 between the sensory nerve^+^ group and the sensory nerve‐ group by RNA‐seq, and GSEA demonstrating gene sets enriched in the sensory nerve^+^ group instead of the sensory nerve^−^ group. *P* < 0.05, *n* = 3 to 4 per group. NES, normalized enrichment score. (D) Representative immunofluorescence labelling of CD68^+^ macrophages, and corresponding quantification of total cell number and CD68^+^ macrophage percentage in the bone defect on day 3, comparing the sensory nerve^‐ ^group with the sensory nerve^+^ group. ^*^ *P < *0.05 by Student's *t* test, *n* = 3 per group. (E) Representative immunofluorescence co‐labelling the expression of CD68 and iNOS in bone defect on day 3 in the sensory nerve^+^ group and the sensory nerve^−^ group, as well as corresponding quantification of iNOS^+^ cell percentage. ^*^^*^ *P < *0.01 by Student's *t* test, *n* = 3 per group. Dots represent individual animals. Data are presented as means ± SD.

Sensory nerves possess the capacity to regulate innate immune responses. To explore the association between sensory nerve function and acute inflammation, we compared transcriptional profiles of healing tissues with intact sensory nerves (sensory nerve^+^) at different time points (Figure 2A). Gene enrichment analysis revealed that sensory perception and leukocyte activation (specifically MCP‐1 production and monocyte aggregates) were significantly enriched on day 3 compared with days 7 and 14, suggesting their functional association during the early phase of bone repair. (Figure 2B). On day 3, neurotransmitter secretion and the neuropeptide signaling pathway are enriched in the group with sensory nerves compared to the group without sensory nerves, supporting the possibility that sensory nerves regulate leukocyte activation through local release of neurotransmitters/neuropeptides in the acute inflammatory phase (Figure 2C).

Osteoclasts originate from mononuclear precursors of the monocyte/macrophage lineage, whose recruitment increases over the days following bone injury. Given prior evidence linking sensory nerve activity to monocyte/macrophage recruitment and osteoclastogenesis, we next analyzed whether sensory innervation modulates macrophage activation and infiltration in bone healing. The results confirmed a significant increase in macrophage (CD68^+^) infiltration in the bone defect without sensory nerve on day 3 compared to the group with sensory nerve (Figure 2D,E). Additionally, the increase of macrophage infiltration was restricted to bone, including bone marrow and endosteum but not found in the distal segment of the transected nerve bundle (Figure S2). The recruited macrophages in sensory denervated bone defect showed strong co‐expression of iNOS (a marker for pro‐inflammatory phenotype of macrophages) (Figure 2F). Taken together, these findings indicate that sensory nerves help control macrophage recruitment and activation through local release of neural molecules in the acute inflammatory phase of bone repair.

### 2.3 CGRP Is Involved in Sensory Nerve‐Mediated Bone Healing

**FIGURE 3:**
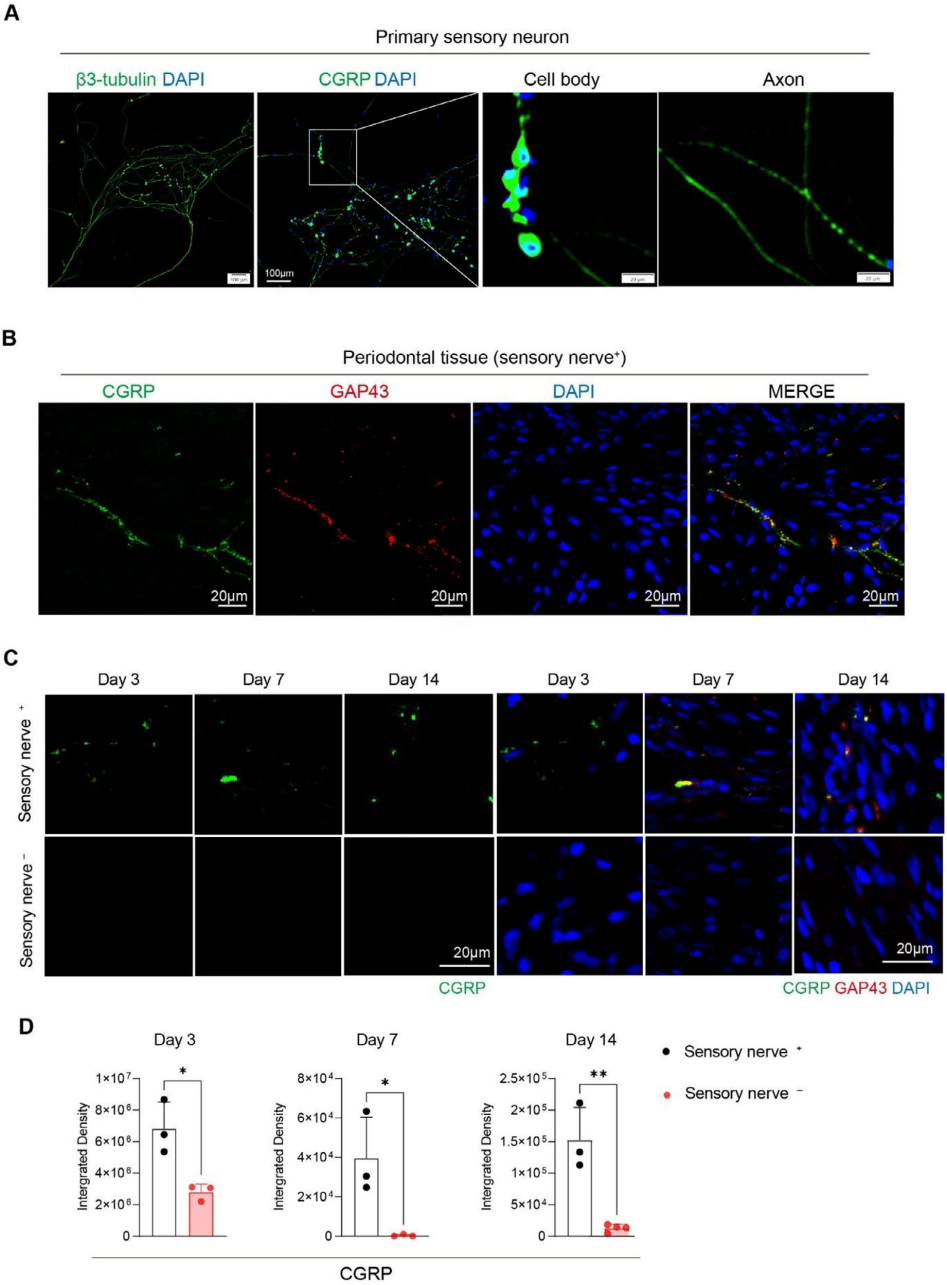
Reduced sensory neuron‐derived CGRP is involved in sensory nerve‐mediated bone healing. (A) Representative immunofluorescence images of primary sensory neurons isolated from dorsal root ganglion (DRG), showing β3tubulin representing neuronal cytoskeleton (left) and CGRP expression (right panel). (B) Representative immunofluorescence images co‐labelling CGRP and GAP43 (a marker for axons) in periodontal tissue adjacent to bone with intact sensory innervation. (C) Representative images of immunofluorescence co‐labelling of CGRP and GAP43 in bone defects on day 3, 7, and 14 post‐surgery from sensory nerve^+^ and sensory nerve^−^ groups. (D) Quantification of CGRP expression in bone defects of each group. ^*^ *P < *0.05, ^**^ *P < *0.01 by Student's *t* test, *n* = 3 to 4 per group. Dots represent individual animals. Data are presented as means ± SD.

Next, we sought to identify the specific sensory nerve–derived signals involved in bone healing. By screening sensory neuron sequencing databases and literature, calcitonin gene‐related peptide (CGRP) emerged as the top candidate to account for our previous observations in vivo, owing to its reported reduction in tissue following sensory denervation and its capability to directly regulate macrophage behaviors reported during soft tissue healing [26]. Firstly, the wide expression of CGRP in primary sensory neurons isolated from dorsal root ganglions (DRG) was observed with strong localization in both cell bodies and axons (Figure 3A). To determine its relevance to bone repair, we tested its expression in periodontal tissue (attaching to bone) and confirmed that it is innervated by CGRP^+^ nerve fibers (co‐labeled with GAP43 as an axonal marker) (Figure 3B). Importantly, in the animal model, CGRP signals were barely detectable in the sensory denervated group compared to controls (Figure 3C). The quantification of CGRP immunofluorescence revealed the statistically significant decrease of CGRP level in sensory nerve^−^ bone defect compared to sensory nerve^+^ group at days 3,7 and 14 (Figure 3D). The correlation of decreased CGRP following sensory denervation indicates CGRP might be involved in sensory nerve‐mediated macrophage activation and bone healing in vivo.

### 2.4 Multiple Functions of Activating Macrophages Are Inhibited by CGRP

**FIGURE 4:**
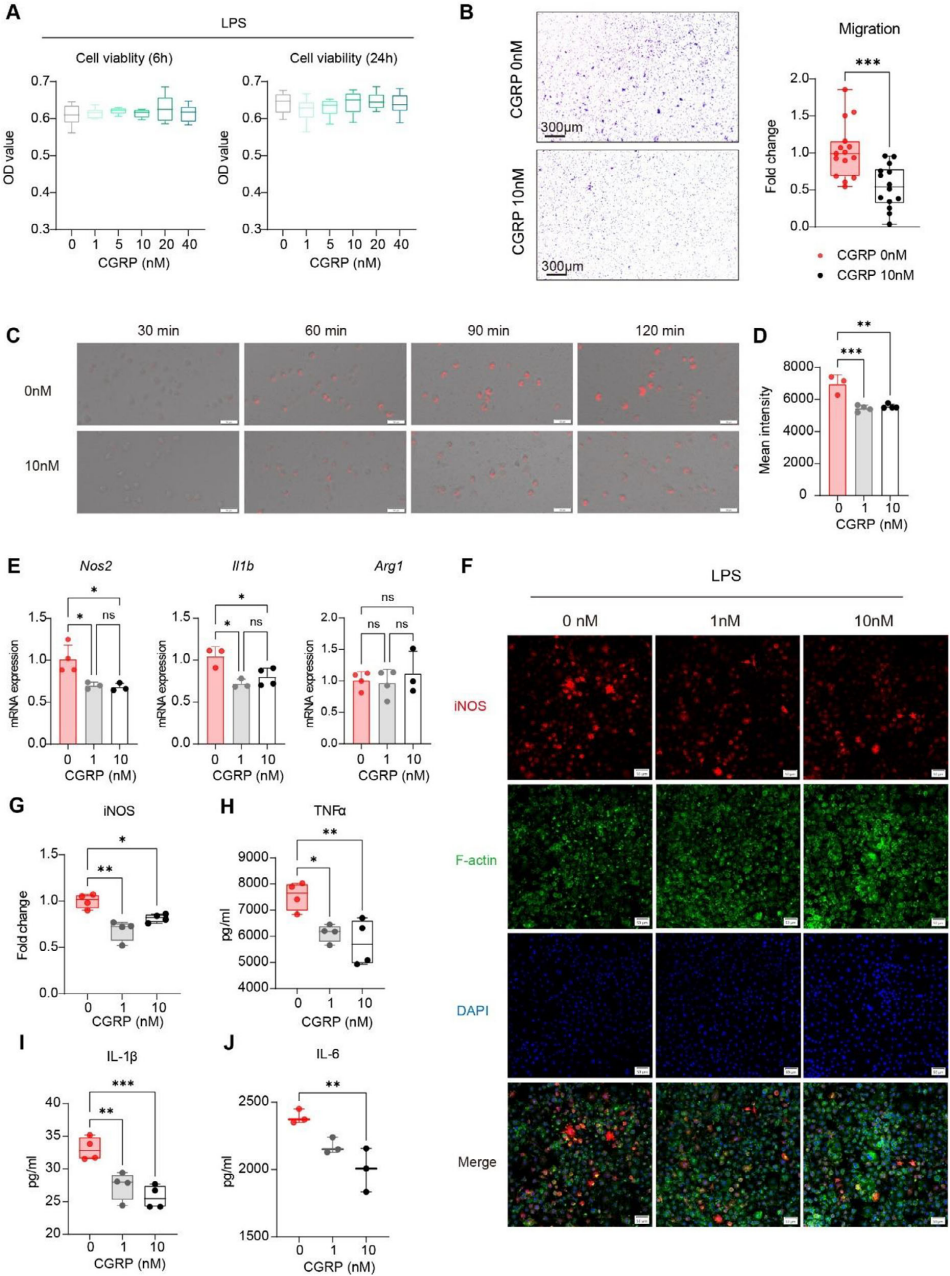
CGRP suppresses macrophage activation, inhibiting migration, phagocytosis, and pro‐inflammatory cytokine production. (A) Cell viability assay of macrophages stimulated with LPS alone or LPS in combination with CGRP gradient (1–40 nm) for 6 and 24 h. Statistical analysis was performed by one‐way ANOVA with Tukey's multiple comparisons test, *n* = 5–6 per group. (B) Migration assay showing representative images of migrated macrophages after 6 h in the presence or absence of CGRP, with corresponding quantification. Statistical analysis was performed by Student's *t* test, ^***^ *P < *0.001, *n* = 14–15 per group. (C) Live‐cell imaging of macrophage phagocytosis showing internalized particles (red signals) at 30–120 min of incubation with or without CGRP. Scale bar, 50µm. (D) Flow cytometry analysis of mean fluorescence intensity of phagocytosed particles after 2 h of incubation with or without CGRP. ^**^ *P < *0.01, ^***^ *P < *0.001 by one‐way ANOVA with Tukey's multiple comparisons test, *n* = 3 to 4 per group. (E) Real‐time PCR analysis of *Il1b*, *Nos2*, and *Arg1* mRNA expression in macrophages after 6 h of LPS stimulation with or without CGRP. ^*^ *P < *0.05 by one‐way ANOVA with Tukey's multiple comparisons test, *n* = 3–4 per group. (F) Confocal images of iNOS expression in macrophages after 24‐h LPS stimulation with or without CGRP. Scale bar, 50µm. (G) Quantification of iNOS expression normalized by cell number. ^*^ *P < *0.05, ^**^ *P < *0.01 by one‐way ANOVA with Tukey's multiple comparisons test, *n* = 4 per group. (H–J) ELISA assay of IL‐1β, TNFα, and IL‐6 in macrophage supernatants after 24‐h LPS stimulation with or without CGRP. ^*^ *P < *0.05, ^**^ *P < *0.01, ^***^ *P < *0.001 by one‐way ANOVA with Tukey's multiple comparisons test, *n* = 3–4 per group. Data presented by the bar graph are means ± SD. Dots represent biological replicates. Data presented by Box and whiskers are Min to Max (show all points if applied).

Given that macrophages express CGRP receptors [26], we next assessed how CGRP influences macrophage responses to stimulation through a series of functional assays. First, it is confirmed that a CGRP gradient ranging from 1 to 40 nm had no effect on macrophage viability after 6‐ or 24‐h LPS stimulation (Figure 4A). To ensure experimental consistency, the working concentrations were determined based on previously published literature employing the same cell type [26]. Since sensory denervation altered macrophage infiltration and activation in vivo, we then examined whether CGRP modulates macrophage migration. Consistent with in vivo results, CGRP was shown to inhibit macrophage migration in vitro (Figure 4B).

Because macrophages are key phagocytes responsible for debris clearance after tissue damage, phagocytosis assay was performed to measure whether CGRP affects their phagocytic activity. Live‐cell imaging of phagocytosis over 2 h revealed weakened fluorescent signals in CGRP group (Figure 4C). Flow cytometry confirmed comparable percentages of positive cells among groups (Figure S3), but the fluorescence intensity per cell was markedly reduced in macrophages exposed to CGRP (Figure 4D), indicating that CGRP diminished phagocytic efficiency rather than the blockade of phagocytosis.

Additionally, CGRP downregulated the transcription of *Il1b, Nos2* (markers for pro‐inflammatory macrophages), while expression of *Arg1* (a marker for anti‐inflammatory macrophages) remained unchanged in activating macrophages (Figure 4E). Correspondingly, at the protein level, CGRP reduced iNOS expression and suppressed secretion of multiple pro‐inflammatory cytokines, including IL‐1β, TNFα and IL‐6 (Figure 4F–J). Taken together, the results suggest that CGRP can inhibit macrophage activation towards a pro‐inflammatory phenotype, aligning with the in vivo observations.

### 2.5 CGRP Induces Transcriptional Alterations in Macrophages Upon Activation

**FIGURE 5:**
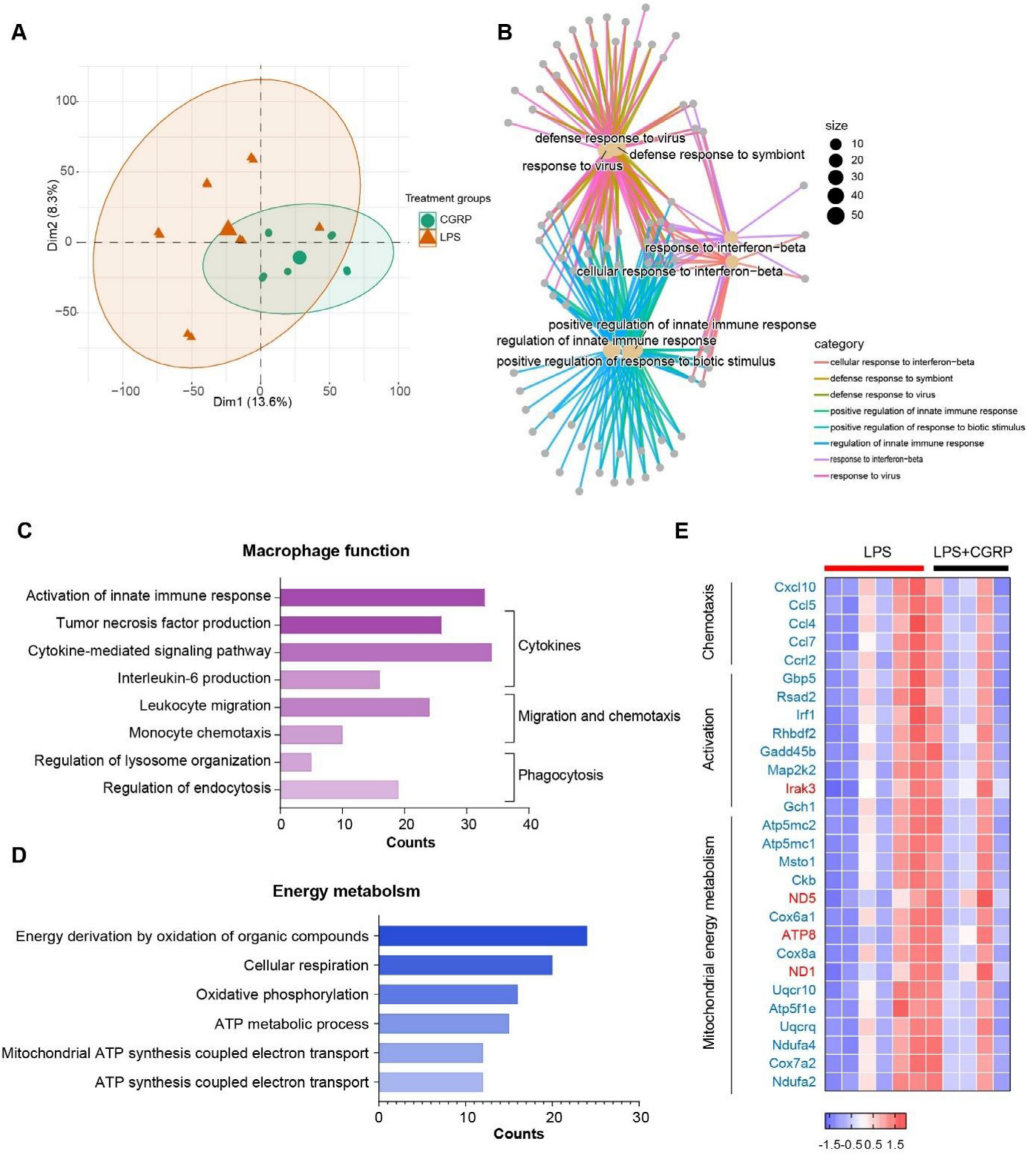
Transcriptomic changes induced by CGRP during macrophage activation. (A) Principal component analysis (PCA) showing transcriptional variation between groups. LPS, macrophages stimulated with LPS alone; CGRP, macrophages stimulated with LPS and CGRP (10 nm). (B) Gene Ontology (GO) enrichment of differentially expressed genes (DEGs) shows a network of enriched biological processes. (C) GO enrichment analysis of DEGs highlighting pathways associated with macrophage functional responses, including cytokine production, migration, and phagocytosis, as listed in the graph. *Q*‐value < 0.05. (D) GO enrichment analysis of DEGs indicates significant enrichment of pathways linked to macrophage energy metabolism, as listed. *Q*‐value < 0.05. (E) Heatmap of representative DEGs identified by comparing LPS+CGRP (10 nm) versus LPS group. Genes are grouped by biological processes as indicated. Blue gene names represent downregulation in the LPS+CGRP group, whereas red gene names represent upregulation relative to LPS.

To investigate the mechanisms of how CGRP suppresses macrophage activation, RNA was extracted from macrophages exposed to LPS alone or in the presence of CGRP. Principal component analysis (PCA) showed samples within the same group clustered together, with separation observed between groups (Figure 5A). A total of 753 differentially expressed genes (DEGs) were identified (adjusted *P* value < 0.05), many of which were associated with immune responses to various stimuli (Figure 5B). Specifically, CGRP profoundly altered biological pathways related to activation of innate immune response, and multiple functions including cytokine production (e.g. TNF production, cytokine‐mediated signaling pathway, IL‐6 production), migration & chemotaxis (e.g. leukocyte migration and monocyte chemotaxis), and phagocytosis (e.g. regulation of lysosome organization and regulation of endocytosis) (Figure 5C), consistent with the in vitro functional assays. Moreover, DEGs were also significantly enriched in energy metabolism pathways, including energy derivation by oxidation of organic compounds, cellular respiration, oxidative phosphorylation (OXPHOS) and adenosine triphosphate (ATP) metabolic process (Figure 5D). Notably, genes associated with macrophage activation, chemotaxis, and mitochondrial energy metabolism, particularly those involved in ATP biogenesis such as multiple mitochondrial complex subunits, were markedly downregulated in the presence of CGRP (Figure 5E).

### 2.6 CGRP Limits Macrophage ATP Generation by Functionally Controlled Mitochondrial Respiration

**FIGURE 6:**
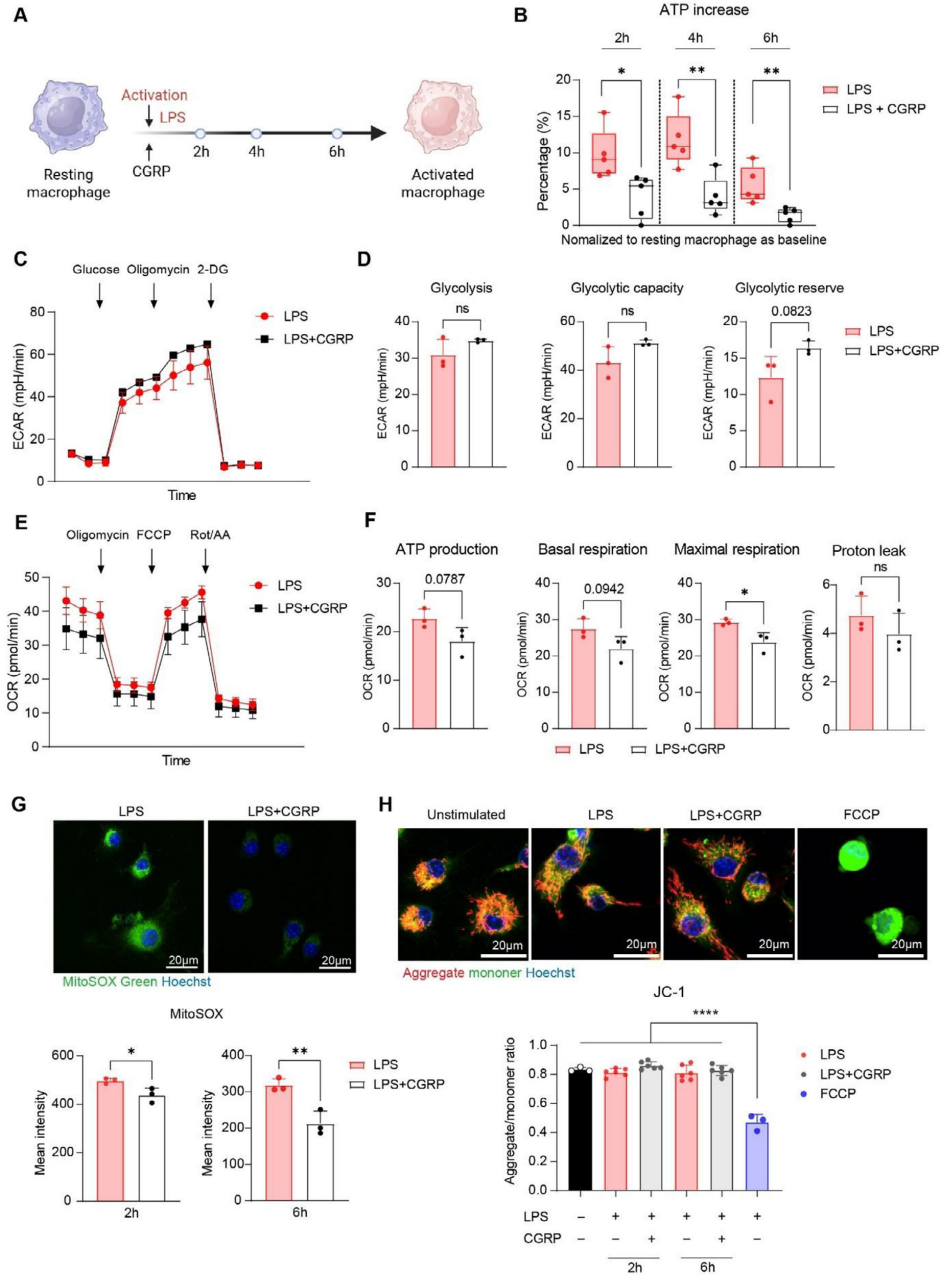
CGRP limits excessive ATP generated by mitochondria in activating macrophages. (A) Schematic illustration of macrophage activation, treatment, and measurement at the indicated time points. (B) Percentage of intracellular ATP increase in macrophages at 2, 4, 6h after LPS stimulation, with or without the presence of CGRP (10 nm). Baseline was defined as intracellular ATP level in resting macrophages (no LPS stimulation). ^*^ *P < *0.05, ^**^ *P < *0.01 by Student's t test, *n* = 5 per group. (C) Dynamic extracellular acidification rate (ECAR) measured during Glycolysis stress test at 4–5 h post‐stimulation (LPS or LPS+10 nm CGRP), with 6‐min recording intervals. Arrows indicate sequential compound injections, annotated in the graph. 2‐DG: 2‐Deoxy‐D‐glucose. (D) Calculated ECAR parameters (glycolysis, glycolytic capacity and glycolytic reserve), normalized to cell number in each well. Statistical analysis was performed by Student's *t* test, *n* = 3 per group. (E) Dynamic oxygen consumption rate (OCR) measured during Mito stress test t 4–5 h post‐stimulation (LPS or LPS+10 nm CGRP), with 6 min recording intervals. Arrows indicate sequential compound injections, annotated in the graph. FCCP, carbonyl cyanide‐p‐trifluoromethoxy phenylhydrazone; Rot/AA, rotenone and antimycin A. (F) Calculated OCR parameters (ATP production, basal respiration, maximal respiration and proton leak), normalized to cell number. ^*^ *P < *0.05 by Student's *t* test, *n* = 3 per group. (G) Representative images of MitoSOX staining of mitochondrial superoxide in activating macrophages with or without CGRP (10 nm), quantified by flow cytometry at 2‐ and 6‐h post‐stimulation. ^*^ *P < *0.05, ^**^ *P < *0.01 by Student's t test, *n* = 3 per group. (H) Representative images of JC‐1 staining showing mitochondrial membrane potential in resting macrophages, activating macrophages with or without CGRP (10 nm) and FCCP‐treated positive control for mitochondrial damage. mitochondrial damage. Quantification was performed by plate reader at 2‐ and 6‐h post‐stimulation. ^****^ *P < *0.0001 by one‐way ANOVA with Tukey's multiple comparisons test, *n* = 3 to 6 per group. FCCP, carbonyl cyanide‐p‐trifluoromethoxy phenylhydrazone. Dots represent biological replicates. Data presented by the bar graph are means ± SD. Data presented by Box and whiskers is Min to Max, showing all points.

As macrophage activation is highly energy‐dependent, our previous results demonstrated that CGRP inhibited macrophage activation, accompanied by decreased cellular activities, raising the possibility that this effect is linked to changes in energy biogenesis. To investigate whether CGRP modulates macrophage ATP production, intracellular ATP levels were measured at different time points during activation (Figure 6A). Compared to the baseline representing cellular ATP level in resting macrophages, both groups showed increased cellular ATP upon stimulation (Figure 6B). However, macrophages exposed to CGRP showed significantly reduced ATP elevation at 2, 4, and 6 h compared to those stimulated with LPS alone. (Figure 6B). Since cells generate ATP primarily through oxidative phosphorylation (OXPHOS, in mitochondria) and glycolysis (in the cytoplasm), we next examined which pathway was affected. Glycolysis stress test and Mito stress test were performed, respectively. Glycolysis stress tests revealed comparable glycolytic rates and glycolytic capacities between the two groups (Figure 6C,D). In contrast, as shown by the Mito stress test, CGRP reduced macrophage basal respiration and oxygen consumption rate for ATP production, while proton leak remained unaffected (Figure 6E,F). These results indicate that CGRP primarily restricts mitochondrial respiration, thereby limiting ATP synthesis during activation. To determine whether impaired mitochondrial function was due to mitochondrial damage, we assessed mitochondrial superoxide production and membrane potential. As a result, CGRP seemed to attenuate mitochondrial superoxide accumulation upon LPS stimulation (Figure 6G; Figure S4), while JC‐1 assays revealed no difference in aggregate/monomer ratios between the two groups, comparable to resting macrophages (Figure 6H). Collectively, these findings suggest that CGRP limited macrophage mitochondrial ATP production during activation, without causing mitochondrial damage.

### 2.7 Macrophages Exposed to CGRP during Activation Have Attenuated Osteoclastogenic Effects

**FIGURE 7:**
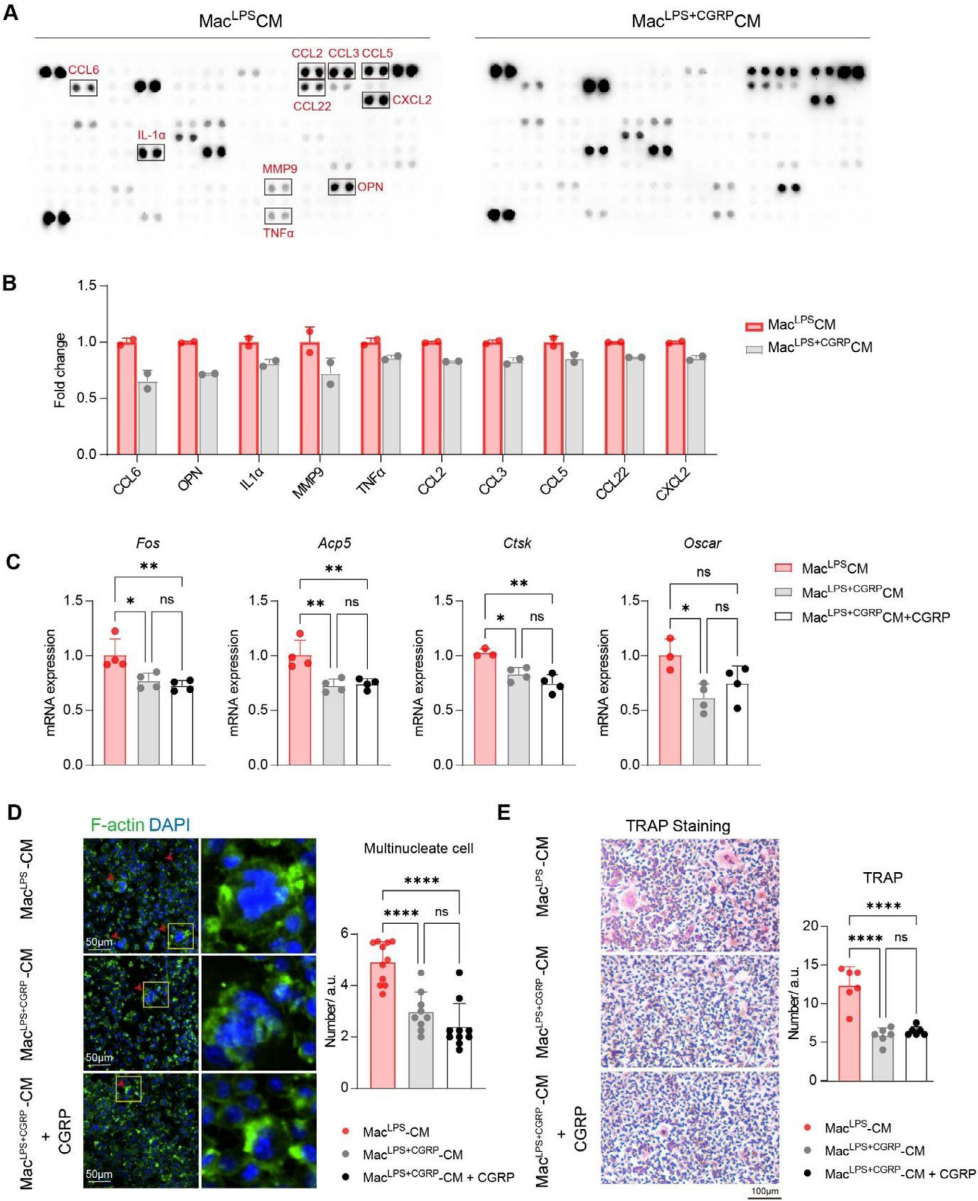
CGRP modulates cytokine production and attenuates osteoclastogenic effect of activated macrophages. (A) Images of cytokine array membrane from macrophage‐conditioned media: Mac^LPS^‐CM (collected from LPS‐treated macrophages) and Mac^LPS+CGRP^‐CM (collected from LPS & CGRP‐cotreated macrophages). Red boxes highlight cytokines with differential expressions between groups, annotated by name on the array. The cytokine layout was identical across both membranes. (B) Quantification of cytokine expression from the arrays. Protein levels were normalized to internal controls on each membrane, using positive controls (located at the upper left, lower left, and upper right corners) and negative controls (lower right corner). *N* = 2 array dots per group. (C) Real‐time PCR analysis of *Fos*, *Acp5*, *Ctsk* and *Oscar* mRNA expression on day 3 of osteoclast differentiation exposed to Mac^LPS^‐CM, Mac^LPS+CGRP^‐CM or Mac^LPS+CGRP^‐CM with direct 10 nm CGRP addition. ^*^ *P < *0.05, ^**^ *P < *0.01 by one‐way ANOVA with Tukey's multiple comparisons test, *n* = 3 to 4 per group. (D) Representative immunofluorescence images of F‐actin on day 7–9 of osteoclast differentiation under the same treatment conditions as in (C). Arrows, multinucleate cells with F‐actin ring. Quantification of multinucleate cells with F‐actin ring in each group is shown. ^****^ *P < *0.0001 by one‐way ANOVA with Tukey's multiple comparisons test, *n* = 9 to 11 per group. (E) Representative images of TRAP staining on day 7–9 of osteoclast differentiation under the same conditions as in (C). Quantification of TRAP^+^ osteoclasts in each group was shown. ^****^ *P < *0.0001 by one‐way ANOVA with Tukey's multiple comparisons test, *n* = 6 per group. Dots represent biological replicates in (C–E). Data throughout are means ± SD.

As detected by cytokine array, CGRP did not dramatically alter the overall secretome of activated macrophages, but reduced the secretion of several cytokines and chemokines, including CCL2, CCL3, CCL5, CCL6, CCL22, CXCL2, TNFα, IL‐1α, and MMP9 (Figure 7A,B). In the context of osteoclast differentiation, conditioned media derived from CGRP‐treated and LPS‐activated macrophages (Mac^LPS+CGRP^‐CM) displayed an attenuated osteoclastogenic effect compared with conditioned media from LPS‐activated macrophages (Mac^LPS^‐CM). Specifically, Mac^LPS+CGRP^‐CM significantly downregulated the expression of osteoclast‐related genes *Fos*, *Acp5*, *Ctsk*, and *Oscar* in pre‐osteoclasts undergoing differentiation (Figure 7C). Functionally, Mac^LPS+CGRP^‐CM treatment resulted in significantly fewer multinucleated cells with F‐actin ring formation and reduced numbers of TRAP^+^ osteoclasts, compared with the control group (Mac^LPS^‐CM) (Figure 7D,E). The effect was further validated in osteoclast differentiation without adding M‐CSF (Figure S5). Moreover, the direct addition of CGRP into osteoclastogenic media supplemented with Mac^LPS+CGRP^‐CM elicited comparable inhibitory effects to Mac^LPS+CGRP^‐CM alone (Figure 7C–E). These results highlight that CGRP modulates macrophage activation and exerts a profound downstream regulatory influence on osteoclast differentiation and bone remodelling.

## 3 Discussion

**FIGURE 8:**
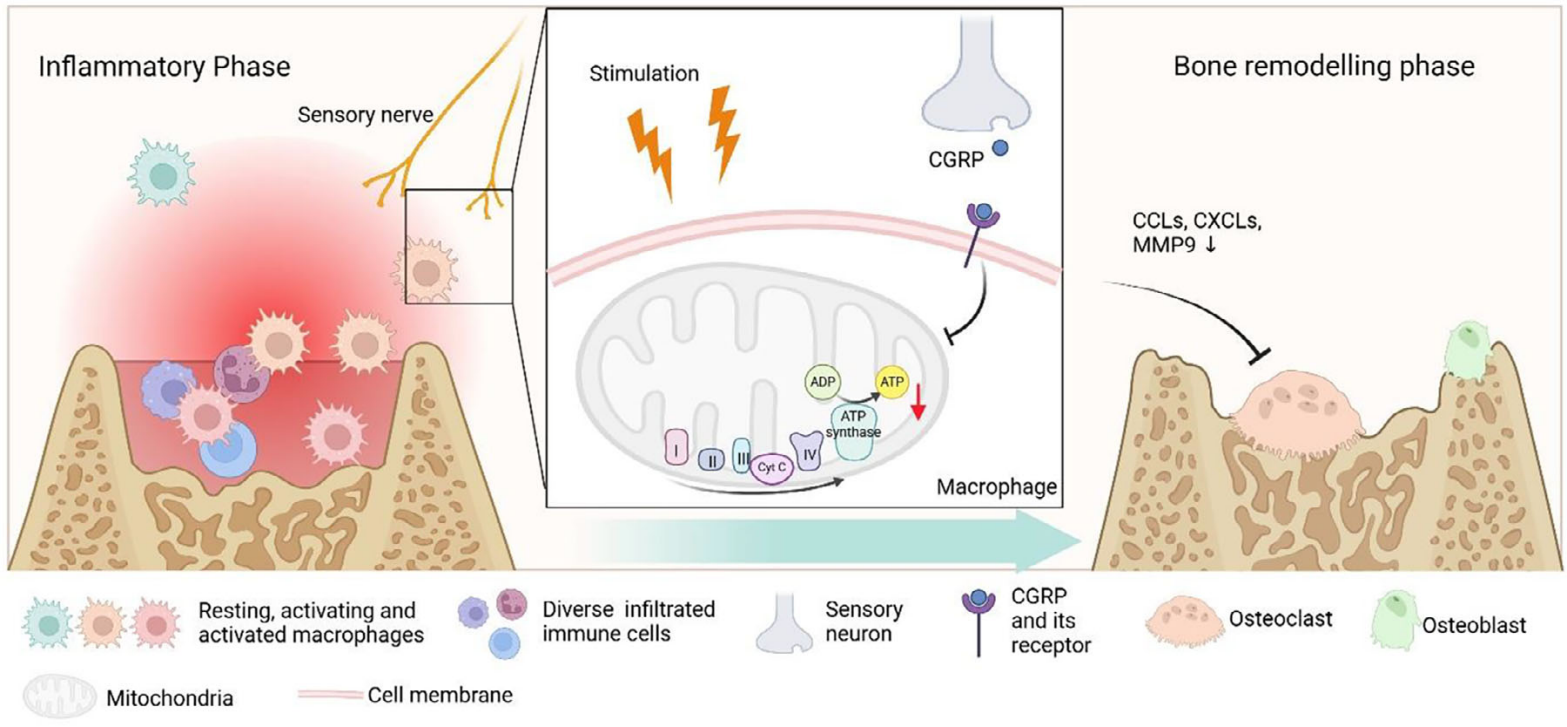
Schematic diagram of the study. The role of sensory nerve‐derived CGRP in macrophage mitochondrial bioenergetics and function is revealed in dynamic bone healing, suggesting new opportunities for immunometabolic therapeutic strategies.

In this research, we highlighted the significant role of sensory nerves in coordinating inflammation and restraining excessive osteoclastogenesis to benefit bone repair. As the powerhouse of many immune cells [27], mitochondrial respiration in activating macrophages is rapidly and effectively controlled by sensory nerve‐derived neuropeptide CGRP, thereby limiting ATP production. CGRP‐mediated macrophage energy metabolism is tightly linked to its activation status and functional output, ultimately exerting a profound regulatory influence on osteoclast differentiation during the bone‐healing cascade (Figure 8).

Recently, accumulating studies suggested that sensory nerves play an active role in bone metabolism, including bone development, bone fracture healing, and pathological bone remodeling (e.g. osteoporosis) [28, 29, 30, 31, 32]. However, a critical knowledge gap persists between neurobiology and bone biology: the mechanisms by which sensory nerves influence osteoimmunology during the sequential dynamics of bone healing remain unclear. Consistent with our findings, the supportive role of sensory innervation in bone repair has been reported in other studies using different models (e.g. nerve transection/chemical injection/gene editing for sensory denervation, as well as bone fracture/socket healing after tooth extraction for bone repair) [30, 33, 34, 35, 36]. The present study further confirmed the association between sensory denervation and increased osteoclast activity, a phenomenon that was also observed in bone fracture healing and tooth movement [32, 37]. To identify the responsible neural molecules, we observed that CGRP is widely expressed in nociceptors across species, suggesting broad and significant biological effects [38, 39]. In support of our findings, CGRP expression has been detected in the inferior alveolar nerve, which innervates the mandible [33, 37]. While CGRP has been reported to directly promote osteogenesis of mesenchymal stem cells (MSCs) and osteoblasts [40, 41]. Our in vivo results similarly revealed the sensory denervation led to the reduced RUNX2 expression (a marker for differentiating osteoblasts) on day 14. As sustained or elevated RUNX2 expression over time is associated with enhanced bone regeneration in this model, the observed decline in RUNX2^+^ cells in the denervated group may reflect impaired osteoblast differentiation at this stage of healing [42, 43]. However, excessive osteoclastogenesis emerged earlier and exhibited more pronounced changes throughout the healing process.

Elucidating the role of sensory nerves in osteoimmunology during the highly dynamic phases of bone repair holds substantial significance. While many studies have focused on the interaction between sensory neurons and bone‐forming cells such as osteoblasts and MSCs [44], bone healing after injury or regenerative therapy is inherently a multi‐phase process orchestrated by inflammation [1]. Consistent with our observation of increased macrophage infiltration in sensory‐denervated bone defects, similar phenomena have also been reported in skin and muscle injury using an alternative sensory nerve ablation model (*Nav1.8^cre^/Rosa26^DTA^* mice) [26]. The sensory neuron‐mediated macrophage infiltration is proven to be CGRP signaling dependent, using an animal model with conditional knockout of the CGRP receptor in myeloid cells [26].

Nevertheless, the role of macrophages in bone repair can be more multifaceted. Beyond their classical functions in debris clearance and shaping the inflammatory microenvironment, macrophages also serve as the precursors of osteoclasts, thereby contributing to osteoclastogenesis both directly (through differentiation into osteoclasts) and indirectly (through secretion of cytokines and chemokines that regulate osteoclast differentiation and survival) [ 45 ]. In the present study, when CGRP reduced both macrophage migration and chemokine production, the regulatory effect on the inflammatory environment and downstream bone resorption could be amplified by the combined effects. Specifically, we proved that CGRP decreased the secretion of several chemokines. The downregulation of these chemokines could have dual consequences: 1) reduced recruitment of macrophage infiltration (fewer osteoclast precursors), and 2) attenuated osteoclastogenic effect, given the known roles of CCLs, CXCLs, TNFα and MMP9 in promoting osteoclast differentiation and survival [45, 46, 47]. However, we did not observe a synergistic effect of the addition of CGRP with Mac^LPS + CGRP^‐CM treatment, which may be attributed to the presence of multiple cytokines within the conditioned medium induced by the treatment of CGRP. This complexity could lead to overlapping mechanisms or saturation of the inhibitory response under in vitro conditions. The role of CGRP in osteoclast formation within a cytokine‐rich microenvironment remains to be fully elucidated.

Notably, our findings revealed that CGRP can rapidly limit macrophage energy production via controlling mitochondrial respiration during classical activation, providing novel insights into neuro‐immune communication mechanisms. A few points that should be highlighted in our research are: 1) the remarkable speed with which CGRP exerts a regulatory effect on limiting macrophage ATP increase, and 2) the extent of control that CGRP achieves without compromising overall cell viability or abolishing fundamental macrophage functions. (e.g. not significantly blocked but comparatively suppressed cell recruitment, phagocytic ability and cytokine production).

Compared to the resting state, macrophage activation relies on energy consumption, establishing a close link between metabolism and function. In response to LPS stimulation, bioenergetic boost is needed to meet acute demands for inflammatory signaling and cytokine secretion [27, 48]. Blocking glycolysis or mitochondrial electron transport chain (ETC) before LPS stimulation can significantly downregulate pro‐inflammatory cytokine secretion by bone marrow‐derived macrophages (BMDMs) [49]. Our results revealed that exposure to CGRP led to rapid control of ATP elevation with limited mitochondrial ATP production. This suggests the involvement of fast‐acting upstream regulatory mechanisms that influence mitochondrial function during the early phase of macrophage activation. Since the binding of CGRP to its membrane receptor is known to activate adenylate cyclase (AC), the rapid effect observed here may be mediated through cAMP as a second messenger that enables mitochondrial modulation, while the mechanisms remain to be elucidated [50]. Importantly, CGRP‐mediated suppression of mitochondrial ATP generation persisted for at least 6 h, as shown by our study, indicating a sustained regulatory influence. The concurrent transcriptional downregulation of multiple mitochondrial complex subunits (complex I, III, IV, V) that directly participate in the electron transport chain (ETC) and OXPHOS suggests a transcriptional basis for long‐term mitochondrial regulation. Together with our observation of reduced cytokine production in conditioned media (48 h post‐stimulation), these findings suggest that CGRP‐mediated regulation of mitochondrial function and macrophage activity may operate both in the acute phase through rapid signaling and in a sustained manner through transcriptional regulation of mitochondrial components.

In the present study, the unchanged mitochondrial membrane potential, along with attenuated mitochondrial superoxide elevation in the CGRP‐treated group, likely reflects a consequence of functionally constrained respiration rather than mitochondrial damage. According to the previous study, this functional modulation of mitochondrial activity may contribute to the attenuation of inflammatory responses not only by restraining mitochondrial ATP production, but also by preventing mitochondrial‐generated inflammatory signals after LPS stimulation [48]. LPS stimulation can induce an early burst of mitochondrial oxygen consumption rate within 6 h [51]. In the meantime, mitochondrial respiration is repurposed towards reactive oxygen species (ROS) production (mainly from Complex I and III), which amplify pro‐inflammatory cytokine production and phagocytosis in BMDMs [48, 52, 53]. Similarly reported in microglia, sustained activation of complex I has been shown to maintain their pro‐inflammatory state due to the increased superoxide production from reverse electron transport (RET) [54]. Therefore, maintaining mitochondrial homeostasis under stress might play a critical role in shaping immune cell phenotypes and functions [55].

Despite changes in mitochondrial activity, macrophage viability remained intact, and their secretome profile was not qualitatively altered, although overall cytokine secretion was reduced. It has been reported that sustained glycolysis is important to support immune cell survival especially when OXPHOS declines [51]. It can help explain the unchanged commitment to glycolysis in our study, which could support cell viability and maintain the cellular functions described above. Collectively, our findings reveal that CGRP induces a rapid but sustained modulation of mitochondrial respiration in classically activated macrophages, exerting subtle yet durable effects with significant biological impact. Specifically, while classically activated macrophages exhibit a strong osteoclastogenic effect through their secretome, activating macrophages exposed to CGRP showed a significantly attenuated osteoclastogenic effect, as shown by in vitro results, consistent with in vivo findings.

While our study shows that sensory neuron‐derived neuropeptide CGRP influences mitochondrial respiration during macrophage activation, the precise mechanisms underlying this regulation remain to be elucidated. Future work should address the specific effects of CGRP on individual mitochondrial complex activities and provide a more comprehensive metabolic profiling of key substrates and intermediates involved in this process. Future studies employing animal tools that provide targeted ablation of CGRP in neurons will be needed to validate and extend our current observations. Finally, our in vitro findings are reported in a murine cell line and are expected to be further validated in primary cells.

In conclusion, we identified that sensory nerve‐derived CGRP regulates macrophage energy production by restraining mitochondrial respiration, attenuating macrophage activation, and inhibiting excessive osteoclastogenesis during the bone‐healing cascade. These findings point to a neuro‐immune‐metabolic axis through which neuronal signals may influence immune cell energy metabolism and inflammatory responses. Although further mechanistic studies are required, our work provides a framework for exploring how sensory nerves contribute to the regulation of inflammation and bone repair and may inspire the development of innovative clinical strategies and regenerative therapies.

## 4 Materials and Methods

### 4.1 Ethical Statement and Animal Information

Animal study was conducted with the approval of the Animal Experimentation Ethics Committee of Nanjing Agricultural University (PZW2022003). Specific pathogen‐free (SPF) male Sprague‐Dawley rats (6–8 weeks old, male) were used for the animal study. Animals were randomly allocated to 2 groups.

### 4.2 Sensory Denervation and Bone Healing Model

Sensory denervation of the mandible was achieved by inferior alveolar nerve transection as previously reported [56]. In brief, after induction of anesthesia via isoflurane inhalation, the segment of the inferior alveolar nerve bundle was exposed before it enters the mandibular canal by surgical manners. Approximately 5mm of the nerve segment was removed to ensure the complete disruption of sensory nerve integrity and function during the period of the experiment. Simultaneously, a periodontal bone defect was created using previously described methods [57]. Briefly, a standardized bone defect size (5mm×2mm×1mm; width*height*depth) was created on the buccal side of the mandible in the molar region. The wound was sutured, and rats were housed in an SPF‐grade animal facility with intraperitoneal injections of penicillin once daily for three consecutive days to prevent postoperative infection. Rats that experienced obvious local infections (e.g. abscesses) were excluded from data analysis.

### 4.3 Micro‐Computed Tomography (Micro‐CT) and Analysis

The harvested mandibles were fixed in 4% paraformaldehyde (PFA) for 48 h and scanned using a high‐resolution micro‐computed tomography system (Bruker micro‐CT, Belgium). Scanning parameters were set at a voxel size of 18 µm. Three‐dimensional reconstruction was performed using CTVol (Bruker, Belgium). A region of interest (ROI) with a fixed size in the bone defect was defined for each layer, avoiding inclusion of tooth root or bone beyond the defect area (15 layers per sample). Quantitative morphometric parameters—including bone volume fraction (BV/TV), trabecular thickness (Tb. Th), trabecular number (Tb. N), and trabecular separation (Tb. Sp) were calculated using CTAn software (Bruker, Belgium).

### 4.4 Histology and Analysis

After fixation in 4% paraformaldehyde for 48 h, rat mandibles harvested at different time points were decalcified in 10% ethylenediaminetetraacetic acid (EDTA) for 10 weeks. Following dehydration in a series of ethanol and xylene, the samples were embedded in paraffin. Sections with a thickness of 5 µm were acquired for H&E staining to evaluate new bone and collagen, and tartrate‐resistant acid phosphatase (TRAP) staining for osteoclast identification. TRAP^+^ cells in the region of the bone defect were calculated. Images were acquired by scanning (Leica AT2 Aperio, Germany). Immunofluorescence staining on tissue sections was performed to determine the expression of Runx2 (1:400, Abcam), CD68 (1:400, Abcam), iNOS (1:400, Abcam), CGRP (1:200, Abcam) and GAP43 (1:400, Abcam). Images were acquired by scanning (Olympus VS200, Japan) or via confocal microscope (Olympus FV3000, Japan). ImageJ was used for quantifying the percentage of positive cells or fluorescence density. Integrated fluorescence intensity was measured in ImageJ using a single fluorescence channel covering the entire bone defect region. For positive cell quantification, positive signals were defined using fixed color‐threshold settings that were consistently applied to all images for the same marker. For each slide, the final value was calculated as the average of measurements from 5–6 randomly selected high‐magnification (60×) fields within the bone defect region. The percentage of positive cells was calculated as the number of positive cells divided by the total number of cells (counted by DAPI).

### 4.5 Macrophage Cell Culture, Stimulation and Cell Viability Test

RAW 264.7 was used for macrophage activation in vitro. The complete medium was Dulbecco's Modified Eagle Medium (DMEM) containing 1% penicillin‐streptomycin (p/s) and 10% FBS. RAW 264.7 were seeded at a density of 2*10^5^ cells/ml unless otherwise stated and allowed to adhere overnight. Cells were then treated with 100 ng/mL lipopolysaccharide (LPS, Escherichia coli 055: B5, R&D Systems) containing 0, 1, 5, 10, 20, or 40 nm of CGRP (Merck). After 6‐ and 24‐h stimulation, cell viability was assessed using Alamar Blue (Thermo Fisher Scientific) according to the kit's instructions. After 3–4 h of incubation, OD value was measured using plate reader (BMG Labtech POLARstar Omega, Germany).

### 4.6 RNA Sequencing

For in vivo samples, healing tissue within the bone defect in different groups at different time points was collected. Samples were tested using the DNBSEQ platform. Reads of low quality, adapter contamination, and high content of unknown bases (N) were removed. After obtaining clean reads, we used HISAT to align the clean reads to the reference genome sequence (Rattus_norvegicus_10116.NCBI.GCF_015227675.2_mRatBN7.2.v2201). Bioinformatic analysis was performed using Dr. Tom platform and RStudio (GSVA).

For in vitro samples, RAW 264.7 were then treated with LPS alone or in combination with CGRP (10 nm) for 6 h. RNA was extracted by PureLink RNA Mini Kit (Thermo Fisher Scientific). Samples were tested using the Illumina NextSeq 2000 platform. Reads of low quality, adapter contamination, and high content of unknown bases (N) were removed using the Cutadapt tool. After obtaining clean reads, we used STAR to align the clean reads to the reference genome sequence (Mus_musculus_mm10/GCF_000001635.20). Bioinformatic analysis was conducted with RStudio (key package info e.g. FeatureCounts, DESeq2, FactoMineR, etc.). Samples that failed quality control were not included in the analysis.

### 4.7 Dorsal Root Ganglion (DRG) Neuron Culture

Primary sensory neurons were provided by Dr. Ju Jin via the sharing of tissue. Briefly, neurons were acquired from mice (postnatal day 7) cervical, thoracic, and/or lumbar DRGs by digesting with 0.25 mg/mL collagenase in PBS supplemented with 25% TripleE. Cells were seeded in DMEM containing 10% FBS, GlutaMAX (Thermo Fisher Scientific), 50 µg/mL gentamicin (Thermo Fisher Scientific) and 5µm cytosine arabinoside (Merck) for 48–72 h. Then the culture medium was replaced by Neurobasal medium supplemented with 1% p/s, 50 ng/mL nerve growth factor (Thermo Fisher Scientific), GlutaMAX (Thermo Fisher Scientific) and B‐27 (Thermo Fisher Scientific) for long‐term culture [58].

### 4.8 Migration Assay

Cell migration was assessed using 24‐well Transwell inserts with 8‐µm pore polycarbonate membranes (Corning, USA). RAW 264.7 (10^5^ cells) are seeded at upper chamber in DMEM. Lower chamber contained conditioned media (dilution 1:1 in DMEM) collected from activated macrophages (24 h stimulation with 100 ng/mL LPS) and supplemented with 5% FBS for the control group. For CGRP group, 10 nm CGRP was added to lower chamber. After 6 h of cell culture, inserts were rinsed in PBS and fixed in 4% PFA for 10min, then stained with 0.5% crystal violet. non‐migrated cells on the upper membrane surface were gently removed. Images of migrated cells were captured with light microscope and cell counting was performed using ImageJ.

### 4.9 Phagocytosis Assay

Commercial kit pHrodo Red S. aureus BioParticles Conjugate (Thermo Fisher Scientific) were used for macrophage phagocytosis assay. RAW 264.7 were seeded in 24‐well plate at the density of 10^5^ cells/mL and incubated overnight. The media were replaced by complete media containing particles at final concentration of 0.1 mg/mL with or without CGRP. Time‐lapse live‐cell imaging was acquired at 30‐min intervals for 2 h (Olympus SpinSR, Japan). For cell flow cytometry, cells were harvested after 2‐h incubation with particles and fixed with 4% PFA for 5min. After washing in PBS, samples were loaded for measurement (BD Biosciences LSRFortessa, USA). Cells without particles served as the negative control, aiding in gating positive cells.

### 4.10 ELISA

Supernatants were collected from macrophages exposed to LPS alone or in combination with CGRP for 24 h. Cell debris was removed by centrifugation at 2 000 rpm for 10 min. Cytokines, including IL‐1β, TNFα, and IL‐6 in the supernatants, were quantified using DuoSet ELISA kits (R&D Systems).

### 4.11 Immunofluorescent Staining and Imaging

Cells were washed in PBS and fixed in 4% PFA for 10min, followed by permeabilization with 0.1%–0.3% Triton X‐100 and blocking with 1% bovine serum albumin (BSA). Primary antibodies used in the study include β3tubulin (1:400, Abcam), CGRP (1:200, Abcam), and iNOS (1:400, Abcam) for overnight incubation at 4°C. Secondary antibodies (1:1000, Abcam) and phalloidin (1:1000, Abcam) were used for 1 h at room temperature. Cells were counterstained with DAPI and examined by a fluorescence microscope (Olympus FV3000, Japan).

### 4.12 ATP Assay

RAW 264.7 cells were seeded in a 96‐well plate one night before the assay. Culture media were replaced with stimulation medium containing LPS/LPS+CGRP. For resting macrophages, complete culture media were used. Cellular ATP was measured after 2, 4, and 6 h of stimulation using an ATP assay kit (Promega), and a positive signal was detected by a plate reader (BMG Labtech POLARstar Omega, Germany).

### 4.13 Glycolysis Stress Test and Mito Stress Test

RAW 264.7 cells were seeded in an assay plate and stimulated with LPS or with LPS in combination with CGRP (10 nm) for 4–6 h. Cells were then processed according to instructions and plates were loaded to monitor ECAR and OCR, respectively, via Seahorse XF Analyzer (Agilent Technologies, USA). Briefly, for the Glycolysis stress test, glucose, oligomycin, and 2‐DG were injected into the well following the order described. For the Mito stress test, oligomycin, FCCP, and a mixture of rotenone and antimycin A were sequentially injected into the wells at final concentrations recommended by the manufacturer's protocol. The value was normalized by cell number in each well.

### 4.14 MitoSOX Staining and Detection

Macrophages were stimulated by LPS or LPS+CGRP (10 nm) for 2, 6 h. After different durations of stimulation, MitoSOX was used to track mitochondrial superoxide with 30min incubation, followed by washing with Live cell imaging solution (Gibco). Live cell imaging was performed for visualization (Olympus SpinSR, Japan), and flow cytometry were conducted for quantification (BD Biosciences LSRFortessa, USA). Cells without MitoSOX dye are included as the negative control. Mitochondrial superoxide was quantified using mean fluorescence intensity. The mean fluorescence intensity of MitoSOX in test groups is calibrated by subtracting the mean fluorescence intensity of the negative control.

### 4.15 JC‐1 Mitochondrial Membrane Potential Assay

Cells were stimulated (LPS or LPS+CGRP) for 2, 6 h. For unstimulated cells, macrophages are cultured in complete media. FCCP (with recommended concentration by manufacturer) was added within 4 h before staining. JC‐1 was added to the culture media and incubated for 10 min. Cells were then washed with PBS. Signals of aggregates and monomers were acquired by live cell imaging (Olympus SpinSR, Japan). Quantification was performed by a plate reader (BMG Labtech POLARstar Omega, Germany).

### 4.16 Cytokine Array

After cell seeding and stimulation (LPS or LPS+CGRP) for 24 h, cells were washed in PBS 3 times. Macrophages were then cultured in DMEM for an additional 24 h. The media were filtered (0.22 µm) and collected as conditioned media for cytokine array and osteoclast differentiation. Proteome Profiler Mouse XL Cytokine Array (R&D) was used for cytokine detection and semi‐quantification following the manufacturer's protocol. The image was acquired by ChemiDoc MP Imaging System (Bio‐Rad, USA).

### 4.17 Osteoclast Differentiation

RAW 264.7 were seeded in a 96‐well plate (10^4^ cells per well). Osteoclast medium containing 25 ng/mL M‐CSF (R&D), 50 ng/mL RANKL (R&D), and 50% of conditioned media (described above) from each group was used to induce osteoclast differentiation, as exhibited in Figure 7. TRAP staining and F‐actin staining were performed 7–9 days after differentiation. For validation of osteoclast differentiation without M‐CSF, the medium remained the same as described, but without the addition of M‐CSF. TRAP staining was performed 6 days after differentiation.

### 4.18 Real‐Time PCR

Gene expression for macrophage activation was tested after 6‐h stimulation, and 3 days for osteoclast differentiation. Total RNA from cells was extracted using TRIzol reagent (Life Technology). Total mRNA was reverse transcribed to cDNA using LunaScript RT SuperMix Kit (New England Biolabs). cDNA templates were amplified with the QuantStudio Real‐ Time PCR machine (Applied Biosystems, USA) using SYBR Green qPCR Master Mix (Life Technologies) and specific primers (Table S1).

### 4.19 Statistical Analysis

Data is presented as mean ± SD. To ensure reproducibility, the results reported are consistent across independent experimental repetitions. For each biological replicate, values were derived from three technical replicates when applicable. Statistical analyses were performed using GraphPad Prism (GraphPad Software, USA). Group comparisons were assessed using Student's *t*‐test or one‐way ANOVA with Tukey's multiple comparisons test, as appropriate. Data with *P* *< *0.05 were marked as statistically significant.

## Author Contributions

J.L. and T.Z. conducted animal surgery and analyzed results. J.L. performed histological staining, in vitro work related to macrophage activation, osteoclast differentiation, ATP assay, metabolic assay, mitochondrial damage test and analysis. J.L. and Y.M. performed the Mito stress test and the Glycolysis stress test, as well as data analysis. K.D. contributed to cell RNA‐seq, data analysis and part of visualization. L.L. contributed to animal work for RNA‐seq and tissue collection. W.G. contributed to part of the imaging work and advised input. J.J. contributed to DRG neuron isolation, shared cells, and provided experiences regarding neuron culture. D.C. contributed assisting with cell work and advised input. F.Y. provided intelligence input and supported conceptualization. J.L., L.X., and Y.X. contributed to the conceptualization, data analysis, and writing of the manuscripts. L.X. and Y.X. provided supervision of the project.

## Conflicts of Interest

The authors declare no conflicts of interest.

## Supporting information




**Supporting File**: advs73731‐sup‐0001‐SuppMat.docx.

## Data Availability

The raw sequencing data generated in this study have been deposited in the Gene Expression Omnibus (GEO) database under accession number GSE314711. Other data that support the findings of this study are available from the corresponding author upon reasonable request.
