# Supplementary material for: Sensory Nerve‐Derived CGRP Controls Osteoclastogenesis by Limiting Macrophage Bioenergetics in Bone Repair
Source: Adv Sci (Weinh). 2026 Mar 1;13(20):e18303. doi: 10.1002/advs.202518303 (PMC13067781; doi:10.1002/advs.202518303)
Supplement: Supplementary file 1 — Supporting File: advs73731‐sup‐0001‐SuppMat.docx. [file ADVS-13-e18303-s001.docx]

**Sensory nerve-derived CGRP controls osteoclastogenesis by limiting macrophage bioenergetics in bone repair**

Jiaying Liu, Ting Zhang, Yuqing Mu, Lili Li, Ju Jin, Kevin J Dudley, Wendong Gao, Donglin Cai, Fuhua Yan^*^, Lan Xiao^*^, Yin Xiao^*^

**Authors and Affiliations**:

J. Liu, Y. Mu, W. Gao, D. Cai, L. Xiao, Y. Xiao

School of Medicine and Dentistry, Griffith University, Gold Coast, Queensland, 4222, Australia

J. Liu, Y. Mu, J. Jin, W. Gao, D. Cai, Y. Xiao

Institute for Biomedicine and Glycomics, Griffith University, Gold Coast, Queensland, 4222, Australia

J. Liu, T. Zhang, L. Li, F. Yan

Nanjing Stomatological Hospital, Affiliated Hospital of Medical School, Institute of Stomatology, Nanjing University, Nanjing, Jiangsu, 210008, China

J. Jin

Clem Jones Centre for Neurobiology and Stem Cell Research, Griffith University, Brisbane, Queensland, 4111, Australia

K. Dudley

Central Analytical Research Facility, School of Biology and Environmental Science, Queensland University of Technology, Brisbane, Queensland, 4000, Australia

***Correspondence**: F. Yan ([yanfh@nju.edu.cn](mailto:yanfh@nju.edu.cn)), L. Xiao ([l.xiao@griffith.edu.au](mailto:l.xiao@griffith.edu.au)) and Y. Xiao ([yin.xiao@griffith.edu.au](mailto:yin.xiao@griffith.edu.au) )

**Keywords**: bone repair, osteoclastogenesis, osteoimmunology, neuro–immune interaction, CGRP, immunometabolism, regenerative medicine

**Supplementary documents:**

**Supplementary Figure 1**

**Supplementary Figure 2**

**Supplementary Figure 3**

**Supplementary Figure 4**

**Supplementary Figure 5**

**Supplementary Table 1**


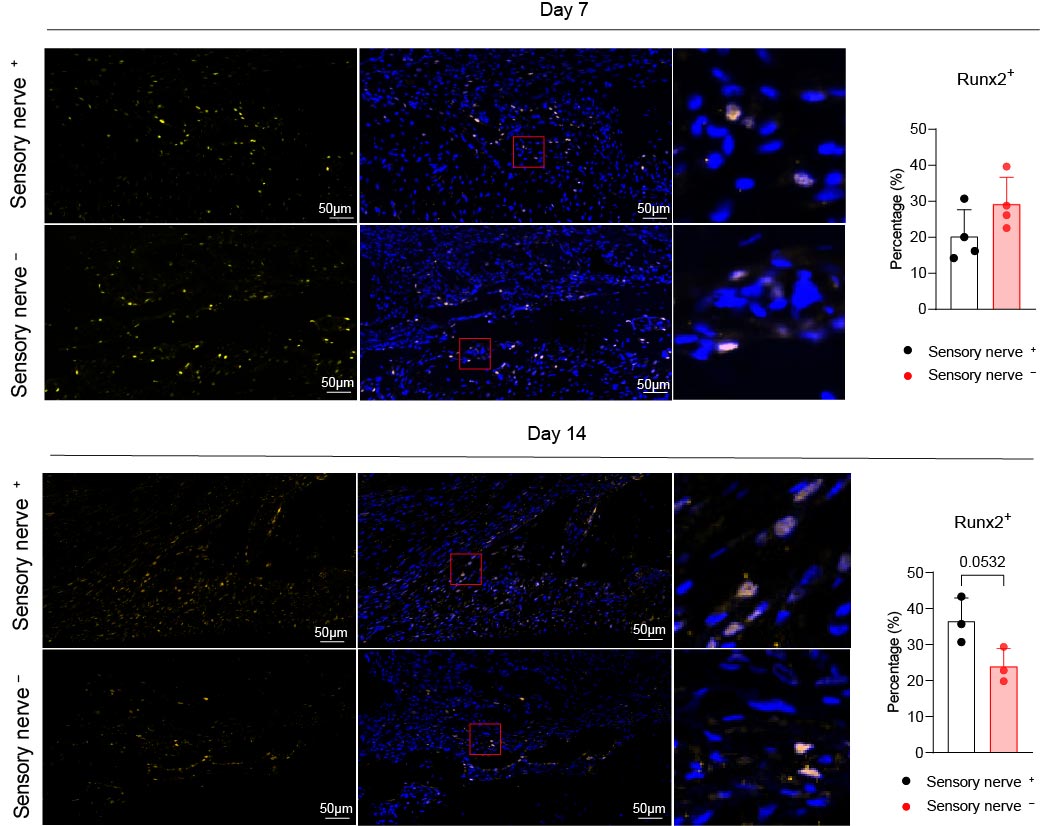


**Supplementary Figure 1**. The effect of sensory innervation on osteoblasts in bone repair. Representative immunofluorescence images labeling Runx2^+^ cells on day 7 and day 14 in the bone defect (comparing sensory nerve^+^ group with sensory nerve^-^ group), as well as corresponding quantification. Statistical analysis was performed by Student’s *t*-test, *n*=3 to 4 per group. Dots represent individual animals. Data throughout are means ± SD.


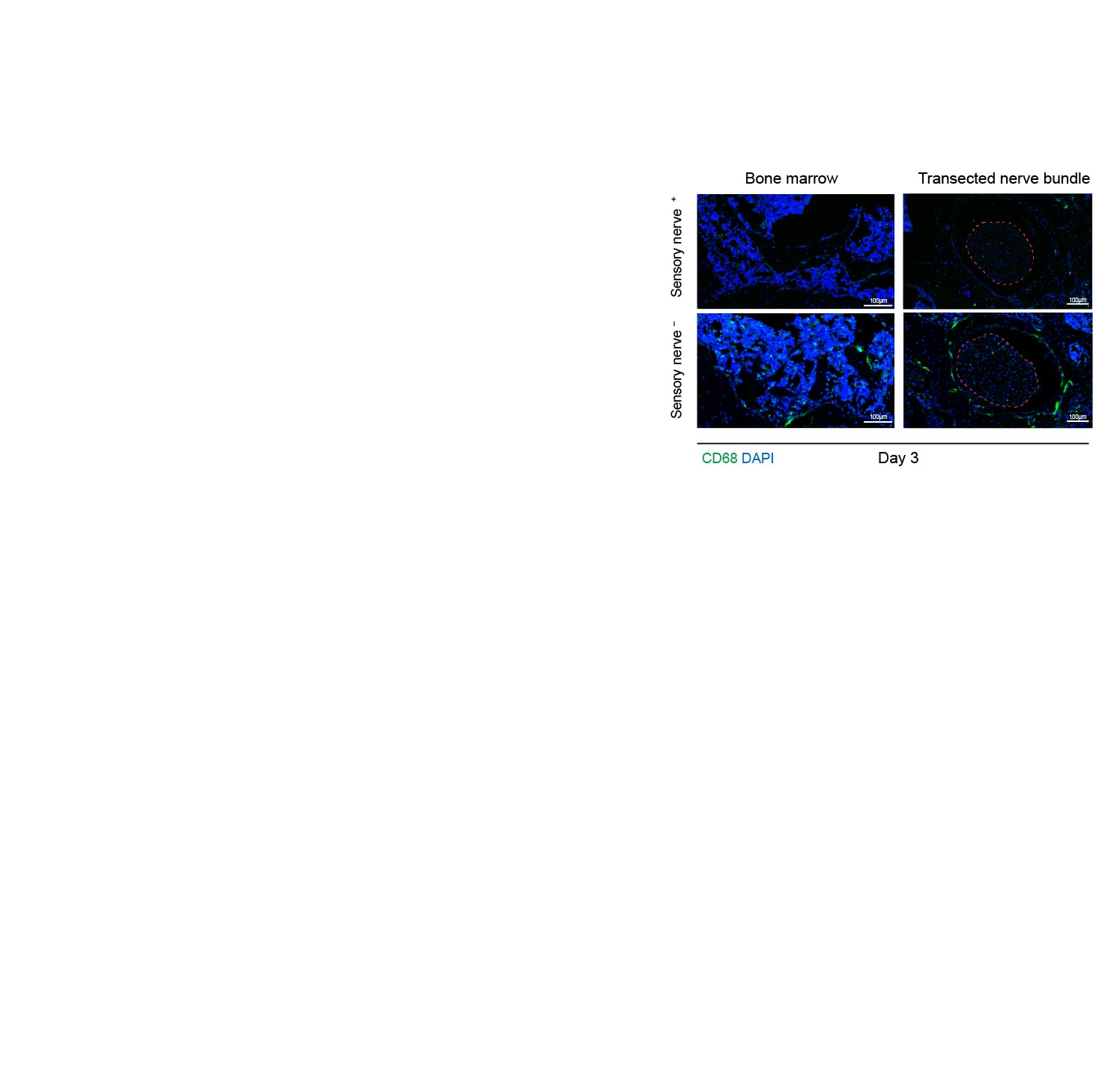


**Supplementary Figure 2**. Increased macrophage infiltration was limited to the bone (not the distal segment of the transected nerve). Representative immunofluorescence images labelling CD68^+^ macrophages on day 3 in bone marrow and endosteum, but not in the distal segment of the transected nerve (circled area) from the sensory nerve group. The sensory nerve^+^ group was provided as a comparison.

**
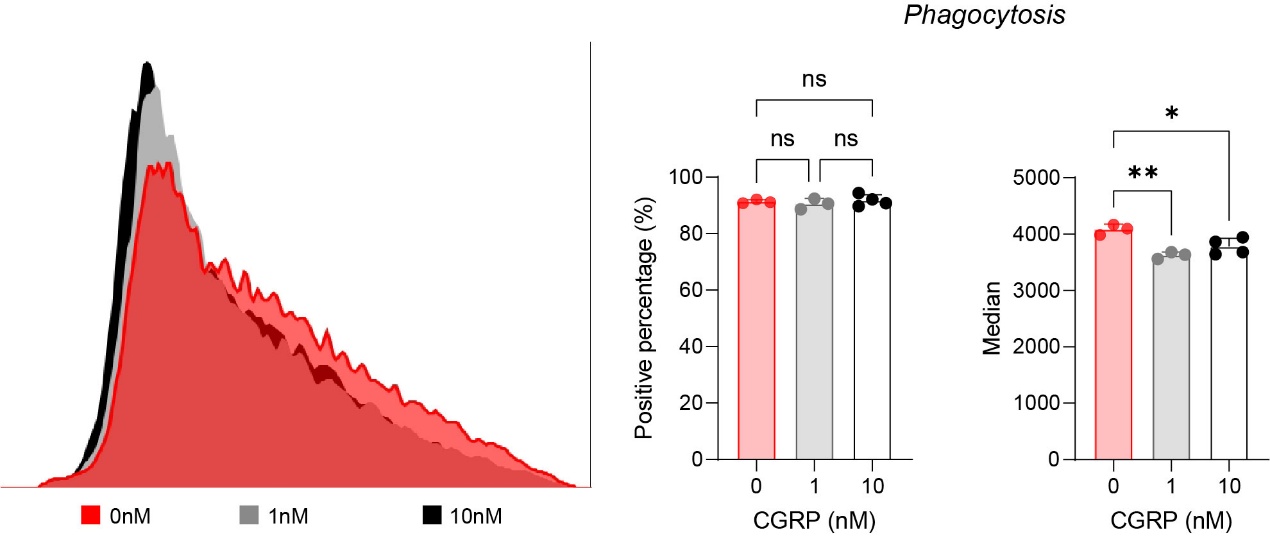
**

**Supplementary Figure 3**. CGRP inhibited phagocytosis efficiency. The histogram shows positive cells from the phagocytosis assay, measured by flow cytometry after 2 hours of incubation with or without CGRP, and the corresponding quantification of positive cell number and median fluorescence intensity in each group. **P*<0.05, ***P*<0.01 by one-way ANOVA with Tukey's multiple comparisons test, *n*=3-4 per group. Dots represent biological replicates. Data throughout are means ± SD.

**
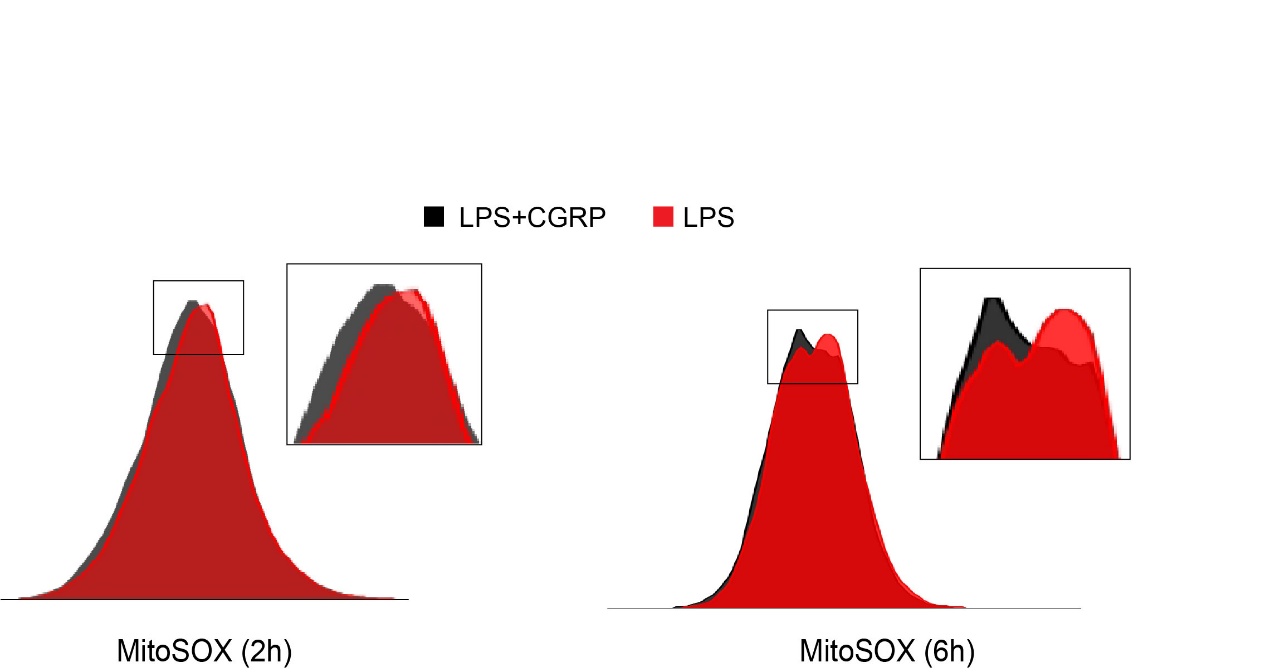
**

**Supplementary Figure 4**. The effect of CGRP on mitochondrial superoxide in response to LPS.. Histogram of MitoSOX labeled mitochondrial superoxide in activating macrophages measured by flow cytometry after 2 and 6 hours of stimulation with or without CGRP.


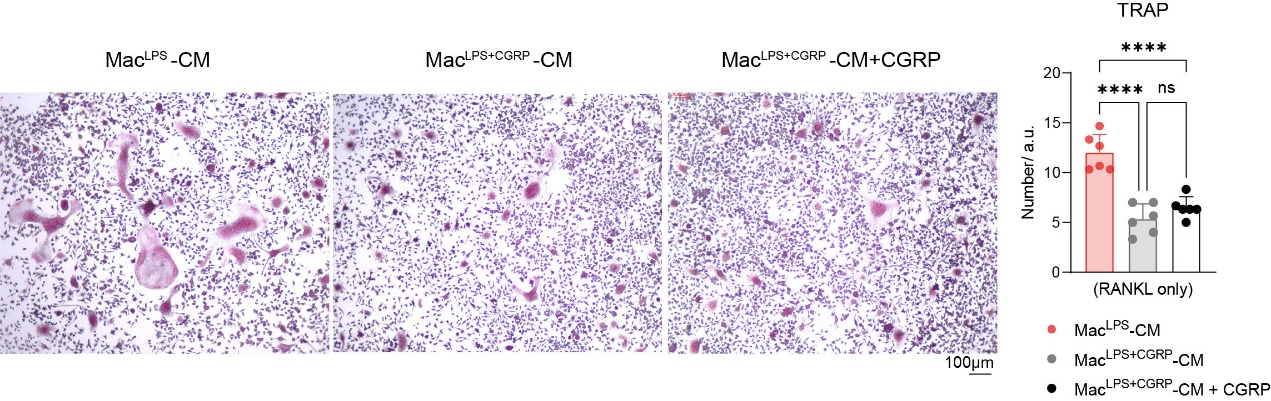


**Supplementary Figure 5.** TRAP staining for osteoclast differentiation without M-CSF. ****P<0.0001 by one-way ANOVA with Tukey's multiple comparisons test, n=6 per group. Dots represent biological replicates. Data throughout are means ± SD.

**Supplementary Table 1.** Primer sequence (Mouse).

| Gene name | Forward | Reverse |
| --- | --- | --- |
| *Gapdh* | GTGTCCGTCGTGGATCTGA | CCTGCTTCACCACCTTCTTG |
| *Il1b* | TGGAGAGTGTGGATCCCAAG | GGTGCTGATGTACCAGTTGG |
| *Nos2* | CACCAAGCTGAACTTGAGCG | CGTGGCTTTGGGCTCCTC |
| *Arg1* | AGAAATTTACAAGACAGGGC | ACTTAGGTGGTTTAAGGTAGTC |
| *Fos* | AAGTGGCAGCCAAGTG | TCAAATCCAGGGAGGCCACA |
| *Acp5* | CGTGCCCTTCGCAACATC | GCATCACTGTCCAGCATAAAG |
| *Ctsk* | CCAGTTTTACAGCAGAGGTGTG | CTTGCTTCCCTTCTGGGTG |
| *Oscar* | GCCCCCTATGTGCTATCACA | ACCAAGGATCCCAGCTTCTC |
